# Measuring physical activity over the life course: cross-cultural validation and measurement properties of the lifetime total physical activity questionnaire for Brazilian older adults

**DOI:** 10.1186/s11556-026-00429-7

**Published:** 2026-08-06

**Authors:** Nara Batista de Souza, Larissa Guimarães Paiva, Christine Friedenreich, Túlio Medina Dutra de Oliveira, Cristino Carneiro Oliveira, Anderson José, Carla Malaguti

**Affiliations:** 1 Department of Physical Therapy for Older Adults, Adults, and Maternal and Child Health, University Feferal of Juiz de Fora, Juiz de Fora, Brazil; 2 Departments of Oncology, University of Calgary, Calgary, Canada; 3 Department of Integrated Health Education, Federal University of Espírito Santo, Vitória, Brazil; 4 Department of Physiotherapy, Federal University of Minas Gerais, Belo Horizonte, Brazil

**Keywords:** Ageing, Physical Activity, Measurement Properties

## Abstract

**Introduction:**

Assessing lifetime physical activity is essential for understanding its cumulative impact on health and ageing; however, no validated instrument is available in Brazilian Portuguese to capture these long-term patterns. The Lifetime Total Physical Activity Questionnaire (LTPAQ) records physical activity across multiple domains and intensities throughout the life course.

**Objective:**

To translate the LTPAQ into Brazilian Portuguese, perform a cross cultural validation and evaluate its measurement properties in a population of Brazilian older adults.

**Methods:**

This methodological study was conducted in two phases: (1) translation and cross-cultural validation following the Beaton guidelines, and (2) measurement properties according to COSMIN standards. The pre-final version was tested in 30 older adults to assess comprehensibility, and the measurement property analyses were conducted in 80 community-dwelling older adults aged 60–80 years. Test-retest reliability, measurement error (standard error of measurement, Bland–Altman limits of agreement), interpretability (minimum detectable difference, floor and ceiling effects), and hypothesis testing and known-groups analyses for construct validity were evaluated. The calendar method was used to improve lifetime activity recall.

**Results:**

The Brazilian Portuguese version of the LTPAQ required minimal cultural adaptation and achieved excellent clarity (content validity index > 0.90).

Test-retest reliability was excellent (ICC = 0.89–0.96), with minimal measurement error (SEM = 1%) and low minimum detectable difference values. Narrow limits of agreement were observed for transportation and exercise/sports, while light-to-moderate activities showed wider variability. Floor effects were present in some domains, but no ceiling effects were identified. No significant correlations were observed between the LTPAQ-Brazil and the EuroQol 5-Dimension questionnaire (EQ-5D), the EuroQol Visual Analogue Scale (EQ-VAS), and the International Physical Activity Questionnaire – Short Form (IPAQ-SF). However, significant differences in lifetime physical activity scores were found according to sex, educational attainment, and socioeconomic status, supporting construct validity.

**Conclusion:**

The LTPAQ-Brazil demonstrated evidence of cross-cultural validity, reliability, measurement precision, interpretability, and construct validity for assessing lifetime physical activity in Brazilian older adults. The instrument enables the characterization of physical activity exposure across the life course and may be useful for epidemiological and healthy ageing research.

**Supplementary Information:**

The online version contains supplementary material available at https://doi.org/10.1186/s11556-026-00429-7.

## Introduction

Physical activity plays an important role in promoting health and preventing disease, serving as a key determinant of well-being over the course of the lifespan [1]. However, many existing physical activity assessment tools measure this behavior at a single point in time or over relatively short periods, thereby failing to capture patterns of physical activity accumulated throughout the life course [2].

Behavioral and lifestyle factors, including physical activity, are of particular interest because they are modifiable through intervention [3]. Participation in physical activities has been consistently reported as beneficial in terms of maintaining physical capacity with advancing age and slowing functional decline [4–7]. However, the association between aging and participation in activities is complex and prone to confusion from reverse causality, as reduced activity can be both a consequence of and a contributing factor to age-related health conditions [8, 9]. This complexity necessitates robust methodological approaches to better understand the long-term impact of physical activity on health, especially in old adults.

Given the challenges of conducting longitudinal cohort studies with follow-up from early life to old age, the use of retrospective assessment methods within existing cohorts may be a viable alternative [10]. In this context, the Lifetime Total Physical Activity Questionnaire (LTPAQ), represents a promising instrument, designed to assess physical activity patterns throughout life, considering occupational, domestic, transportation and leisure activities. Already validated for various contexts [11–18], the LTPAQ has been used to study the relationship between physical activity throughout life and the prevention of diseases such as cancer [11, 14, 15], cognitive disorders [16], depression [18] and age-related health problems [17].

One important feature of the LTPAQ is the use of calendar recall, which organizes key life milestones, medical history and activity records to support memory retrieval. The LTPAQ is applied using cognitive interviewing techniques, which help participants to reconstruct more accurately their lifetime physical activity patterns. However, the LTPAQ has not yet been translated and cross culturally validated for Brazilian older adults. Cultural and socioeconomic differences, as well as variations in lifestyle habits, highlight the need for context-specific validation of assessment tools.

We hypothesized that the Brazilian Portuguese version of the Lifetime Total Physical Activity Questionnaire (LTPAQ-Brazil) would demonstrate satisfactory reliability, interpretability, and construct validity. Specifically, we could expect, as measures of construct validity, weak positive correlations (*r* < 0.30) between the LTPAQ-Brazil and measures of current physical activity and health status, given the differences in the time frame of the constructs assessed. We also hypothesized that lifetime physical activity scores would be higher among individuals with higher educational attainment and socioeconomic status and would differ between men and women. Therefore, this study aimed to cross culturally validate the LTPAQ for use with Brazilian older adults and assess its reliability, interpretability and construct validity, allowing an assessment of lifetime physical activity patterns in this population.

## Methods

This methodological study aimed to evaluate the cross-cultural validity and measurement properties of the LTPAQ.The study was approved by the Institutional Ethics Committee and all participants signed the informed consent forms. Participants were recruited by means of personal invitations within the community, social media postings, and as companions in the waiting room of the Physical Therapy Department at the University Hospital.

The research was conducted in two phases: (1) translation and cross-cultural validation, and (2) evaluation of measurement properties, including reliability, interpretability and construct validity of the LTPAQ. Phase 1 followed the Guidelines for the Process of Cross-Cultural Adaptation of Self-Report Measures¹⁹, while Phase 2 adhered to the methodological standards of the COSMIN initiative (Consensus-based Standards for the selection of health Measurement Instruments) [19, 20].

### Study sample

Phase 1 involved two translators, two back-translators and an expert committee comprising five members, as well as 30 participants in the pre-test [21]. For Phase 2, sample size estimation followed COSMIN recommendations²^0^, as being ≥ 100 participants considered adequate; 50–99 good; 30–49 moderate; and < 30 considered small. The recruited sample was balanced by sex.

### Eligibility criteria

The same eligibility criteria were applied in both phases, however, Phase 1 participants were not invited to take part in Phase 2 in an attempt to avoid bias resulting from prior exposure to the instrument. Individuals considered suitable for inclusion were: apparently healthy older adults of both sexes, aged 60–80 years, with self-reported independence in daily activities and no chronic health conditions likely to impair functional capacity. Individuals with controlled hypertension (without beta-blockers) and controlled diabetes mellitus were included.

The exclusion criteria were as follows: cognitive impairment identified by a score ≤ 4 on the Six Item Screener (SIS) [22], unstable cardiovascular disease, neurological disorders, musculoskeletal conditions preventing independent mobility, a diagnosis of chronic respiratory disease, hospitalization, or the need to withdraw from the study.

### Phase 1 – translation and cross-cultural adaptation

After obtaining authorization from the original author and copyright holders, the process was divided into five stages:

- Stage 1 – Initial Translation: The English version of the LTPAQ was translated into Brazilian Portuguese by two independent, bilingual translators. One translator was familiar with the concepts being examined, ensuring conceptual equivalence, whereas the other was naïve to the subject matter, ensuring language appropriateness and comprehensibility for the target population. Conceptual rather than literal translation was prioritized, resulting in Translation 1 (T1) and Translation 2 (T2).
- Stage 2 – Synthesis of Translations: T1 and T2 were compared and merged into a single version (T1-2), incorporating the best elements.
- Stage 3 – Back Translation: T1-2 was independently back-translated into English by two bilingual translators blinded to the original instrument and with no involvement in the study. The back-translations (BT1 and BT2) were compared with the original LTPAQ to identify discrepancies, producing a consolidated English version (BT1-2), which was submitted to the developer of the original LTPAQ for review and approval.
- Stage 4 – Expert Committee Review: Five bilingual health professionals independently reviewed each item for clarity, cultural relevance and conceptual equivalence. Disagreements were resolved by consensus. The content validity index (CVI) [23] was used using a 4-point Likert scale, considering scores “3” or “4” as agreement. Minimum acceptable CVI is 0.80, and preferably ≥ 0.9023.
- Stage 5 – Pre-Final Version Testing: The culturally adapted pre-final version was applied to 30 older adults with varying educational backgrounds. To enhance recall, participants used the LTPAQ memory-aid calendar, which organizes major life milestones, medical and reproductive history, and records of activity. Trained interviewers applied cognitive interviewing techniques to improve accuracy in recalling lifetime activity patterns. Participants provided verbal feedback regarding clarity, comprehension, wording, and cultural relevance of each item during interviews. Items were considered comprehensible when participants reported no difficulty understanding the questions or response options. Suggestions and doubts raised during the interviews were discussed by the research team and included when considered relevant for cultural adaptation.

Following these steps, the research team and the original authors reviewed the entire process documentation and agreed the adaptation was successfully completed.

Phase 2 began with the assessment of participants’ cognitive function using the SIS [22], quality of life using the EQ-5D and EQ-VAS questionnaires [24], and current levels of physical activity using the short version of the International Physical Activity Questionnaire (IPAQ) [25]. Following these assessments, the LTPAQ was administered via an in-person interview, during which calendar recall was used to assist participants with retrieval of information about their lifetime physical activity habits. This calendar also serves as a reference for data collection during the interview. Initially, participants’ life milestones, such as educational background, family and occupational history, and relevant medical events, were recorded, followed by the age at which each physical activity began and finished, as well as the weekly frequency and average duration for each activity. Activities were classified into three domains: occupational, sports and leisure time.

Physical activity patterns were recorded encompassing information that included the start and end age, number of months per year, days per week, and hours per day for each activity, in order to determine the frequency and duration of these activities. Participants also provided a description of the activities, allowing classification as sedentary (limited to occupational activities), light, moderate or vigorous. The LTPAQ assigns activity intensity using standardized codes and MET (metabolic equivalent) values from the Compendium of Physical Activities, an internationally recognized resource that classifies physical activities according to their energy expenditure and facilitates standardized assessment across epidemiological studies. The method defines intensity according to the ratio of the energy cost of the activity to resting energy expenditure (1 MET = 3.5 mL O₂·kg⁻¹·min⁻¹), in which intensities are categorized as light (< 3 METs), moderate (3–6 METs) or vigorous (≥ 6 METs).

To calculate the total hours dedicated to activities performed throughout the participants’ lives, each category of activity (occupational, domestic and exercise/sports) and intensity level (light, moderate and vigorous) were considered separately. The calculation was carried out employing the following formula:

Total hours in the category over lifetime.

∑= (age at termination - age at initiation) × frequency × hours per day in the activity.

To determine the average number of hours spent per week by each individual, we used the following formula:

Average hours per week in each category.

Total hours in the category over lifetime ÷ age ÷ 52.

The LTPAQ-Brazil generates estimates of lifetime physical activity exposure, expressed as average hours per week. Scores are calculated separately for activity domains (occupational, household, transportation, and exercise/sports) and intensity levels (light, moderate, and vigorous activity). Higher scores indicate greater cumulative participation in physical activity throughout life. In the present study, the primary outcome was the average number of hours of physical activity per week over a lifetime, calculated for each activity category and used as the basis for all measurement property analyses, including assessments of reliability, measurement error, and construct validity. The LTPAQ was chosen due to its unique capacity to capture detailed lifetime physical activity patterns across multiple domains, which is not typically achieved by conventional short-term recall questionnaires. Its calendar recall approach improves memory retrieval and allows for a more comprehensive estimation of habitual activity over the life course, making it particularly valuable in populations where lifetime exposure to physical activity is a relevant determinant of health outcomes.

### Statistical analysis

Data analysis was performed using IBM SPSS software (version 22.0; SPSS Inc., Chicago, IL, USA). The distribution of continuous variables was assessed using the Shapiro-Wilk test. Normally distributed data were expressed as mean and standard deviation (SD), while non-normally distributed data were reported as median and interquartile range (IQR). Categorical variables were summarized by frequency and proportion. A significance level of *p* < 0.05 was adopted for all analyses. The LTPAQ-Brazil measurement properties evaluated were ceiling and floor effects, reliability (standard error of measurement, test-retest reliability), interpretability and construct validity [19].

### Reliability

The LTPAQ-Brazil was re-administered after an interval of 7–14 days, which is sufficient to minimize recall bias while reducing the likelihood of true changes in the construct being measured, thereby providing an appropriate timeframe for test–retest reliability assessment [19].Test-retest reliability was analyzed using an intraclass correlation coefficient (ICC) and 95% confidence intervals. An ICC < 0.50 was considered poor, 0.50 ≤ ICC ≤ 0.75 as moderate, 0.75 ≤ ICC ≤ 0.90 as good, and ICC > 0.90 as excellent reliability [26]. The absolute-agreement type was calculated using the mean weekly physical activity scores for each category obtained from the test and retest assessments, based on a two-way mixed-effects model. The two-way model was chosen to analyze variability between participants and between tests, while the absolute agreement type was selected to assess the consistency of measurements between the two time points.

Agreement was assessed using the standard error of measurement (SEM), calculated as SEM = SD × √(1 − ICC), where SD represents the standard deviation of the baseline scores and ICC the test–retest reliability coefficient. Lower SEM values indicate lower measurement error and greater precision of the instrument [19].

For the Bland–Altman analysis, the mean difference between test and retest scores (bias) and the 95% limits of agreement (mean difference ± 1.96 × SD of the differences) were calculated and visually inspected to identify systematic bias and the extent of agreement between repeated measurements.

### Interpretability

The minimum detectable difference (MDD) was assessed to reflect the variability associated with individual scores on the LTPAQ-Brazil and to specify the magnitude of change that the measurement should demonstrate. The MDD with 95% confidence was calculated using the formula MDD = 1.96 × √2 × SEM^21^. Ceiling and floor effect were calculated based on the proportion of participants who scored the highest (ceiling) and lowest (floor) hours on the LTPAQ-Brazil, and considered present if 15% or more of the respondents achieved the maximum or minimum scores [20].

### Construct validity - hypothesis testing

Following COSMIN recommendations [20], predefined hypotheses regarding the expected direction and magnitude of the associations were established. We hypothesized weak positive correlations (*r* < 0.30) between the average hours per week of activity over a lifetime and measures of current physical activity (IPAQ) and health status (EQ-5D and EQ-VAS), given that these instruments assess different timeframes of assessment and related but distinct constructs.

### Construct validity - known-groups

Known-groups validity was determined using the Mann-Whitney *U* test to compare men and women, socioeconomic levels and levels of education. The groups with higher and lower socioeconomic status were determined according to the Brazilian Economic Classification Criterion (CCEB) [27] developed by ABEP (Associação Brasileira de Empresas de Pesquisa), where the higher socioeconomic status was represented by groups A, B1 and B2, while the lower status was composed of groups C1, C2 and D-E. For the definition of socioeconomic level, we considered classes A and B as high, C as medium, and D-E as low. Participants who had completed a higher education degree were classified as having a higher educational level, whereas those who had completed high school or less were classified as having a lower educational level.

We hypothesized that differences in lifetime physical activity scores would be observed between men and women and across educational and socioeconomic groups, reflecting distinct opportunities and patterns of physical activity participation throughout life [19].

### AI-assisted language editing

During manuscript preparation, ChatGPT (OpenAI) was used for English language editing and assistance with sentence rephrasing. The AI tool was not used for data generation, statistical analysis, interpretation of results, or scientific decision-making. All AI-assisted content was critically reviewed and approved by the authors, who take full responsibility for the final manuscript.

## Results

### Phase 1 - translation and cultural adaptation

The translation and cross-cultural adaptation process used to develop the final version of the LTPAQ-Brazil can be found in Supplement S2. Minor discrepancies between the initial translations were identified mainly in semantic wording and expressions related to activity intensity. These differences were resolved by consensus during the synthesis stage. The back-translation versions showed conceptual equivalence with the original instrument and did not require substantial modifications. During Phase 1, a total of 30 participants were evaluated. The expert panel suggested that the intensities was clearly described in the questionnaire, using the following categories: 1 - Sedentary, 2 - Light, 3 - Moderate, 4 - Vigorous. After this modification, the questionnaire was re-evaluated, and the content validity index was 0.94.

Twenty-eight participants (93.3%) had no difficulty understanding the instrument and did not suggest making any changes. Two participants (6.7%) asked questions, which were clarified and understood but no modification was suggested. Additionally, all dimensions were answered, satisfactorily understood, and applicable to the 30 participants included in this phase. The pre-final Brazilian Portuguese version of the LTPAQ did not require any further changes. The final version of the LTPAQ-Brazil is available in Supplement S1. The demographic and clinical characteristics of the participants are summarized in Table 1.

**Table 1:** Characteristics of the study sample

Variables	Phase 1 – Pre-test sample (*n* = 30)	Phase 2 – Measurement properties sample (*n* = 80)
Age, years	69.0 ± 6.4	68.5 ± 5.2
Female gender, n (%)	18 (60)	40 (50)
HTN, n (%)	16 (53)	19 (24)
DM, n (%)	8 (27)	12 (15)
Regular physical activity, n (%)	7 (23)	46 (58)
Smoking		
Smoker	2 (7)	3 (4)
Ex-smoker	9 (30)	15 (19)
Non-smoker	19 (63)	24 (30)
Education level, n (%)		
Illiterate	0 (0)	3 (4)
Completed elementary school	2 (7)	30 (38)
Completed middle school	9 (30)	11 (14)
Completed high school	13 (43)	17 (21)
Completed university degree	6 (20)	19 (24)
Marital status, n (%)		
Single	0 (0)	8 (10)
Married	21 (70)	46 (58)
Widowed	6 (20)	20 (25)
Divorced	3 (10)	6 (8)
Socioeconomic level (ABEP), n (%)		
High	4 (14)	13 (16)
Medium	12 (40)	35 (44)
Low	14 (46)	32 (39)

*Abbreviations*: *HTN* Hypertension, *DM* Diabetes mellitus, *ABEP* Brazilian Association of Market Research Companies

### Phase 2 - assessment of measurement properties

**Fig. 1:**
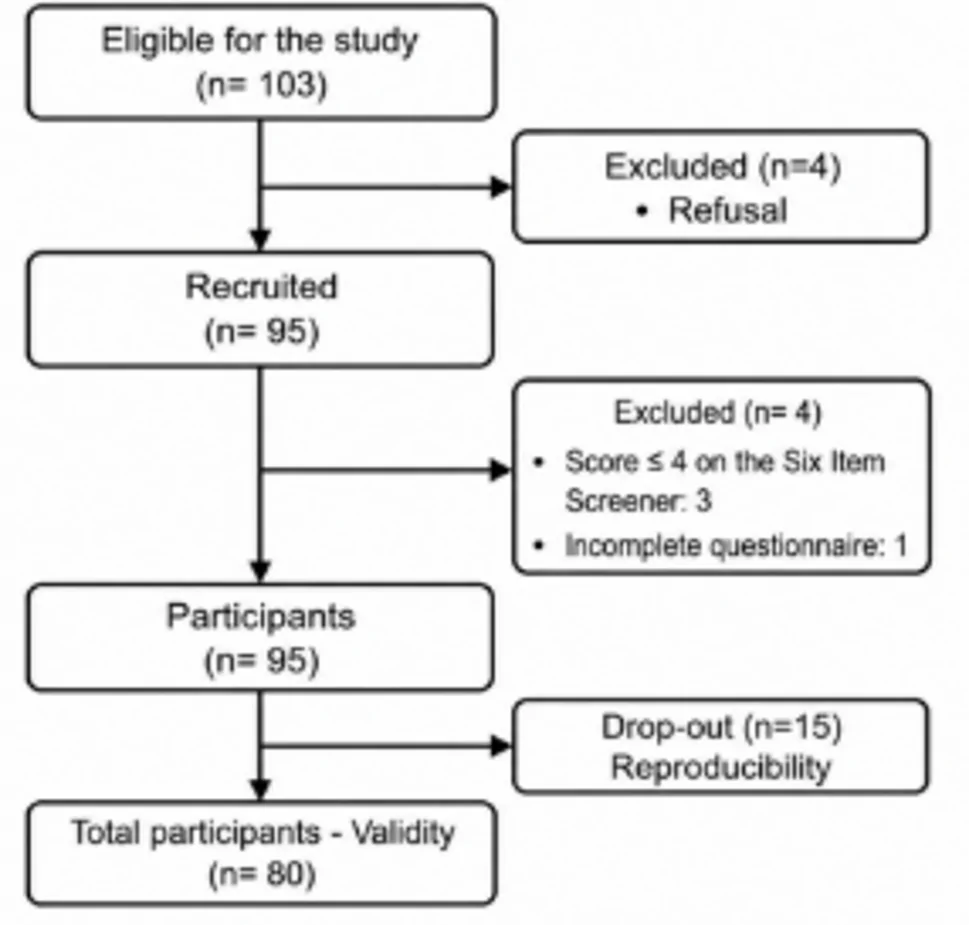
Flowchart of the study sample of measurement properties

During Phase 2 of the study, 103 individuals were recruited. Of these, 4 declined to participate and 19 participants were excluded during assessments. Reasons for exclusion were: scored less than or equal to 4 on the Six Item Screener (*n* = 3), did not complete the questionnaire due to inconsistency in the information (*n* = 1), and were absent during the reproducibility assessment (*n* = 15). Thus, the validity sample comprised 80 participants (Fig. 1), with a mean age of 68.5 ± 5.2 years, of whom 50% were female. The characteristics of the sample are detailed in Table 1.

**Table 2:** Test-retest reliability of the LTPAQ-Brazil

Category of activity	Test	Retest	SEM (%)	MDD	Mean Difference	Limits of agreement	ICC [95% CI]	Floor effect *n* (%)	Ceiling effect *n* (%)
Occupational activity	0.45 ± 0.33	0.45 ± 0.32	1	0.09	-0.01	-0.28–0.27	0.95 [0.92–0.97]	11 (14)	1 (1)
Household activity	0.33 ± 0.31	0.32 ± 0.29	1	0.08	-0.01	-0.25–0.23	0.95 [0.93–0.97]	26 (32)	1 (1)
Exercise and sports	0.03 ± 0.02	0.03 ± 0.03	1	0.008	0.00	-0.02–0.03	0.93 [0.89–0.96]	20 (25)	1 (1)
Transportation activity	0.01 ± 0.01	0.01 ± 0.01	1	0.003	0.00	-0.01–0.01	0.96 [0.93–0.97]	34 (42)	3 (4)
Light activity	0.27 ± 0.26	0.32 ± 0.31	1	0.15	0.02	-0.10–0.52	0.89 [0.83–0.93]	21 (26)	1 (1)
Moderate activity	0.50 ± 0.45	0.45 ± 0.38	1	0.17	-0.05	-0.48–0.38	0.92 [0.88–0.95]	22 (28)	1 (1)
Vigorous activity	0.06 ± 0.14	0.05 ± 0.14	1	0.003	-0.00	-0.18–0.18	0.89 [0.84–0.93]	63 (79)	1 (1)

*Abbreviations*: *ICC* Intraclass Correlation Coefficient, *95% CI* 95% Confidence Interval, *SEM* Standard Error of Measurement, *MDD* Minimum Detectable Difference

The mean scores for each category of activity in the total LTPAQ-Brazil test and retest are presented in Table 2. The primary outcome used in all measurement property analyses was the average hours per week of activity over a lifetime. In the first assessment, the average hours per week over a lifetime ranged from 0.01 ± 0.01 to 0.50 ± 0.45, and from 0.01 ± 0.01 to 0.45 ± 0.38 in the second assessment. The test-retest reliability ranged from good to excellent across all dimensions, with coefficients ranging from 0.89 to 0.96 (95% CI). The standard error of measurement was considered very good (1%) across all categories of the LTPAQ-Brazil. These findings indicate low measurement error and high score stability over time.

The Bland-Altman analysis assessed the agreement between the hours of physical activity performed throughout the participants’ lifetimes, and showed small differences in the domains of the LTPAQ-Brazil between the test and retest. Mean differences were close to zero across all activity domains, indicating minimal systematic bias between assessments. The limits of agreement were narrow in categories such as transportation and exercise/sports, indicating high levels of agreement. In contrast, for light-to-moderate activities, the intervals were wider, suggesting greater individual variability. However, overall agreement remained satisfactory across all domains. The Bland-Altman plots illustrating this comparison can be seen in the Supplementary material (Figure S1).

The minimum detectable difference for the LTPAQ-Brazil ranged from 0.003 h to 0.170 h across the categories. No ceiling effect was detected in the questionnaire dimensions, but the floor effect was detected in all activities except occupational (Table 2).

The known-group validity analysis revealed significant differences between men and women in several domains of the LTPAQ-Brazil, specifically in occupational activity, household activities, exercise and sports, light activity, and vigorous activity (Table 3).

**Table 3:** Comparison between sexes by average hours per week of activity over a lifetime

Category of activity	Female (*n* = 40)	Male (*n* = 40)	*p* value	Effect size
Occupational activity	0.56 ± 1.00	0.57 ± 0.34	0.022*	0.01
Household activity	0.58 ± 0.41	0.21 ± 0.29	< 0.001*	1.04
Exercise and sports	0.03 ± 0.42	0.04 ± 0.37	0.049*	0.02
Transportation activity	0.35 ± 0.12	0.19 ± 0.25	0.078	0.82
Light activity	0.41 ± 0.33	0.28 ± 0.31	0.006*	0.41
Moderate activity	0.74 ± 1.02	0.43 ± 0.40	0.068	0.40
Vigorous activity	0.01 ± 0.06	0.12 ± 0.22	< 0.001*	0.68

*p < 0.05

Additionally, participants with different socioeconomic status showed significant differences in occupational activity, transportation activity, exercise and sports, and moderate activity. Individuals with lower socioeconomic status reported higher levels of occupational activity (0.68 ± 0.90 vs. 0.36 ± 0.28 h/week; *p* = 0.022), transportation activity (0.04 ± 0.11 vs. 0.01 ± 0.01 h/week; *p* = 0.014), and moderate activity (0.73 ± 0.94 vs. 0.33 ± 0.31 h/week; *p* = 0.004), whereas those with higher socioeconomic status reported higher participation in exercise and sports (0.47 ± 0.05 vs. 0.28 ± 0.29 h/week; *p* = 0.018) (Table S1).

Similarly, significant differences according to educational attainment were observed for occupational activity, transportation activity, light activity, moderate activity, and vigorous activity. Participants with lower educational attainment reported higher levels of occupational activity (0.65 ± 0.83 vs. 0.29 ± 0.17 h/week; *p* = 0.003), transportation activity (0.34 ± 0.10 vs. 0.00 ± 0.00 h/week; *p* = 0.048), moderate activity (0.68 ± 0.87 vs. 0.28 ± 0.27 h/week; *p* = 0.006), and vigorous activity (0.09 ± 0.19 vs. 0.00 ± 0.01 h/week; *p* = 0.030), whereas participants with higher educational attainment reported higher levels of light activity (0.50 ± 0.36 vs. 0.30 ± 0.31 h/week; *p* = 0.004) (Table S2). Consistent with our predefined hypotheses for the known-groups construct validity, significant differences in lifetime physical activity scores were observed according to sex, educational attainment, and socioeconomic status of the LTPAQ-Brazil.

To assess construct validity, the LTPAQ-Brazil was compared with the IPAQ, EQ-5D, and EQ-VAS. As hypothesized, only weak correlations were observed between the LTPAQ-Brazil domains and measures of current physical activity and health status (*r* = -0.219 to 0.204). None of these correlations reached statistical significance, suggesting that lifetime physical activity exposure, as measured by the LTPAQ-Brazil, represents a construct that differs from current physical activity and current health status (Table 4).

**Table 4:** Correlations between the LTPAQ-Brazil, IPAQ (short form) and EQ-5D

Independent Variable	EQ-5D *R*	EQ-VAS *R*	IPAQ *R*
Occupational activity	-0.219	0.015	-0.038
Household activity	-0.048	0.005	-0.061
Exercise and sports	0.204	0.184	-0.178
Transportation activity	-0.167	0.074	-0.128
Light activity	-0.026	0.189	-0.041
Moderate activity	-0.218	-0.049	-0.044
Vigorous activity	-0.029	-0.017	-0.068

*Abbreviations*: *IPAQ* International Physical Activity Questionnaire, *EQ-5D* EuroQol 5-Dimension Questionnaire, *EQ-VAS* EuroQol Visual Analogue Scale

## Discussion

This study translated and cross culturally validated the LTPAQ into Brazilian Portuguese and assessed its measurement properties for use in older adults in Brazil. The findings confirm that the LTPAQ-Brazil exhibits adequate measurement properties, including the absence of systematic bias, high test–retest reliability, low measurement error, satisfactory agreement, evidence of construct validity, and negligible ceiling effects. Although floor effects were observed in some activity domains, these likely reflect the activity patterns of the study population rather than a limitation of the instrument. These results support its application for assessing physical activity patterns across the lifespan in both clinical and research settings.

The translation and cross-cultural validation process required only minor semantic adjustments from the expert committee, with no additional modifications required after pre-testing. Pre-testing confirmed excellent clarity, reflected in high content validity index scores, and indicated that the instrument was easily understood by the target population. The interviewer-administered format and the use of calendar recall techniques likely contributed to the high reliability observed in this study. Shephard et al. reported that interviewer-administered questionnaires produce more accurate results than self-administered ones [28]. The average length of interview was about 40 min, with rest breaks as needed, and the process was well accepted by the participants.

The LTPAQ-Brazil demonstrated good to excellent test–retest reliability across all activity domains, with ICC values ranging from 0.89 to 0.96. These findings indicate that the instrument provides highly stable estimates of lifetime physical activity exposure when administered on separate occasions. Measurement properties evaluation revealed excellent reliability, consistent with previous findings from the Persian version of the LTPAQ [29]. The reliability coefficients found are also comparable to, and in some cases higher than, those reported in previous studies evaluating lifetime physical activity questionnaires. For example, Chasan-Taber et al. reported ICC values ranging from 0.78 to 0.87 for different lifetime physical activity domains [30]. Therefore, the reproducibility of the LTPAQ-Brazil appears consistent with the measurement performance reported in other studies and cultural adaptations of lifetime physical activity questionnaires. In addition to the high ICC values, the low standard error of measurement observed across all domains suggests minimal measurement error. Furthermore, the Bland–Altman analyses demonstrated mean differences close to zero between test and retest assessments, indicating the absence of relevant systematic bias. Although wider limits of agreement were observed for light and moderate activities, likely reflecting greater variability in recalling these activities over long periods of life, overall agreement remained satisfactory across all domains. From a measurement perspective, the combination of high reliability, low measurement error, and satisfactory agreement provides evidence that the LTPAQ-Brazil produces reproducible estimates of lifetime physical activity exposure. These measurement properties are particularly relevant for observational studies investigating associations between long-term physical activity patterns and health outcomes in older adults.

Measurement error was minimal (SEM = 1%), an important methodological contribution since many previous validations of the LTPAQ did not report SEM [11–16, 29]. The low minimum detectable difference indicates that relatively small differences in lifetime physical activity scores can be distinguished from measurement error, supporting the precision of the instrument when interpreting repeated assessments.

Floor effects were observed in specific activity domains, mostly in vigorous activities, indicating that a substantial proportion of participants reported no engagement in these activities across their lifetime. Although these effects exceeded the standard 15% threshold, this finding is not unexpected in older populations, as participation in specific types of physical activity may be strongly influenced by sex roles, occupational history, socioeconomic circumstances, and opportunities for leisure-time exercise throughout life. For instance, the high proportion of men reporting zero household activity and women reporting no participation in structured exercise and sports reflects the established sociocultural patterns characteristic of the generations represented in this sample. Consequently, these domain-specific floor effects likely represent a true absence of the behavior rather than a limitation in the instrument.

Furthermore, ceiling effects were virtually absent in all domains, suggesting that the LTPAQ-Brazil retains adequate discriminative capacity for evaluating individuals with higher levels of lifetime physical activity exposure. The Bland–Altman analysis indicated narrow limits of agreement for transportation and exercise/sports, reflecting high consistency, but broader limits for light-to-moderate activities, possibly due to greater variability in these behaviors over the life course [29].

Construct validity analyses revealed the expected differences between the sexes: men reported higher participation in sports, exercise and vigorous activities, while women reported greater involvement in domestic and light activities, consistent with the Persian version of the LTPAQ [29] and previous epidemiological patterns [31, 32]. Variations in educational and socioeconomic status were also in alignment with known trends [31, 32], where individuals with higher levels of education and income were more involved in structured and recreational activities, and those with lower levels reported more occupational or transportation-related activity. These differences underscore the influence of social determinants on lifetime physical activity and highlight the need for tailored public health strategies.

The weak and non-significant correlations observed between the LTPAQ-Brazil and the IPAQ, EQ-5D, and EQ-VAS were consistent with our predefined hypotheses. Although all instruments are related to physical activity and health, they assess different constructs and timeframes. The LTPAQ-Brazil measures cumulative physical activity exposure across the lifespan, whereas the IPAQ assesses recent physical activity and the EQ-5D and EQ-VAS reflect current health status. Therefore, strong associations between these measures were not expected. Instead, weak correlations were anticipated, as lifetime physical activity exposure may not necessarily correspond to current activity levels or current health status in older adults. These findings reinforce that the LTPAQ-Brazil captures a distinct dimension of physical activity behavior, reflecting cumulative exposure over decades rather than short-term or current experiences.

This study has its limitations. Lifetime recall questionnaires are susceptible to recall bias, especially in older adults, although cognitive screening was performed to minimize this risk. Validation against short-term recall or objective measures is inherently limited, as these methods do not capture lifetime patterns. Additionally, results cannot be generalized to individuals aged over 80, and the non-probabilistic sample from a single region may limit external validity. Future research should evaluate other aspects of construct validity, test the instrument in diverse populations, and explore its predictive capacity for health outcomes.

## Conclusion

In summary, the LTPAQ-Brazil demonstrates robust measurement properties and fills an important gap in terms of the tools available for assessing lifetime physical activity in Brazilian older adults. Its use can enhance epidemiological research, guide clinical decision-making and inform interventions by providing a comprehensive view of physical activity history over the life course. This perspective enables the development of more individualized, effective strategies for health promotion and healthy ageing.

## Supplementary Information

Supplementary Material 1.

## Data Availability

The datasets generated and/or analyzed during the current study are available from the corresponding author on reasonable request.

## Abbreviations

ABEP: Brazilian Association of Market Research Companies
BT1, BT2 and BT1-2: back-translations
CI: Confidence Interval
COSMIM: Consensus-based Standards for the selection of health Measurement Instruments
CVI: Content Validity Index
DM: Diabetes mellitus
EQ-5D: EuroQol 5-Dimension Questionnaire
EQ-VAS: EuroQol Visual Analogue Scale
HTN: Hypertension
ICC: Intraclass Correlation Coefficient
IPAQ: International Physical Activity Questionnaire
IQR: Interquartile Range
LTPAQ: Lifetime Total Physical Activity Questionnaire
LTPAQ-Brazil: Brazilian version of the Lifetime Total Physical Activity Questionnaire
MDD: Minimal Detectable Difference
MET: metabolic equivalent
SD: Standard Deviation
SEM: Standard Error of Measurement
SIS: Six Item Screener
T1, T1-2 and T2: translations
