# Supplementary material for: Measuring physical activity over the life course: cross-cultural validation and measurement properties of the lifetime total physical activity questionnaire for Brazilian older adults
Source: Eur Rev Aging Phys Act. 2026 Aug 6;23:33. doi: 10.1186/s11556-026-00429-7 (PMC13543574; doi:10.1186/s11556-026-00429-7)
Supplement: Supplementary file 1 [file 11556_2026_429_MOESM1_ESM.docx]

**Supplements**

Figure S1: Agreement in the number of hours between the test and retest by Bland-Altman analysis. The continuous line represents the mean differences between the tests, and the dotted lines correspond to the 95% confidence interval. A) Occupational activities, B) Household activities, C) Transportation activities, D) Exercises and sports, E) Light activities, F) Moderate activities, G) Vigorous activities. Abbreviations: UL: Upper limit, MD: Mean difference, LL: Lower limit

Table S1: Comparison between socioeconomic levels by average hours per week of activity over a lifetime

| **Activity categories** | **Lower socioeconomic level**  **(n = 50)** | **Higher socioeconomic level**  **(n = 30)** | **p value** | **Effect size** |
| --- | --- | --- | --- | --- |
| Occupational activity | 0.68 ± 0.90 | 0.36 ± 0.28 | 0.022* | 0.48 |
| Household activity | 0.42 ± 0.35 | 0.35 ± 0.47 | 0.141 | 0.17 |
| Exercise and sports | 0.28 ± 0.29 | 0.47 ± 0.05 | 0.018* | 0.91 |
| Transportation activity | 0.04 ± 0.11 | 0.01 ± 0.01 | 0.014* | 0.38 |
| Light activity | 0.30 ± 0.32 | 0.41 ± 0.34 | 0.099 | 0.33 |
| Moderate activity | 0.73 ± 0.94 | 0.33 ± 0.31 | 0.004* | 0.57 |
| Vigorous activity | 0.09 ± 0.19 | 0.02 ± 0.10 | 0.061 | 0.46 |

*p < 0,05

Table S2: Comparison between education levels by average hours per week of activity over a lifetime

| **Activity categories** | **Lower education level (n=61)** | **Higher education level (n=19)** | **p value** | **Effect size** |
| --- | --- | --- | --- | --- |
| Occupational activity | 0.65 ± 0.83 | 0.29 ± 0.17 | 0.003* | 0.60 |
| Household activity | 0.37 ± 0.35 | 0.46 ± 0.53 | 0.406 | 0.20 |
| Exercise and sports | 0.33 ± 0.36 | 0.43 ± 0.49 | 0.131 | 0.23 |
| Transportation activity | 0.34 ± 0.10 | 0.00 ± 0.00 | 0.048* | 4.80 |
| Light activity | 0.30 ± 0.31 | 0.50 ± 0.36 | 0.004* | 0.59 |
| Moderate activity | 0.68 ± 0.87 | 0.28 ± 0.27 | 0.006* | 0.62 |
| Vigorous activity | 0.09 ± 0.19 | 0.00 ± 0.01 | 0.030* | 0.67 |

*p < 0,05

SUPLEMENT S1 – versão final do LTPAQ-Brasil
